# Supplementary material for: Boolean modeling of breast cancer signaling pathways uncovers mechanisms of drug synergy
Source: PLoS One. 2024 Feb 23;19(2):e0298788. doi: 10.1371/journal.pone.0298788 (PMC10889607; doi:10.1371/journal.pone.0298788)
Supplement: S1 Table — (DOCX) [file pone.0298788.s002.docx]

**Table S1.** The missense mutations collected from five cell lines available in Cell Model Passports and DepMap.

| Cell lines | HGNC name | Model name | Amino acid mutation | Effect | Status |
| --- | --- | --- | --- | --- | --- |
| BT-549 | TP53 | P53 | p.R249S | Likely Loss-of-function | 0 |
| BT-549 | PTEN | PTEN | p.V275fs*1 | Likely Loss-of-function | 0 |
| MCF-7 | GATA3 | GATA3 | p.D336fs*17 | Likely Loss-of-function | 0 |
| MCF-7 | PIK3CA | PI3K_c | p.E545K | Gain-of-function | 1 |
| MDA-MB-231 | NF2 | NF2 | p.E231* | Likely Loss-of-function | 0 |
| MDA-MB-231 | NF1 | NF1 | p.T467fs*3 | Likely Loss-of-function | 0 |
| MDA-MB-231 | TP53 | P53 | p.R280K | Loss-of-function | 0 |
| MDA-MB-231 | KRAS | RAS_i | p.G13D | Gain-of-function | 1 |
| MDA-MB-468 | TP53 | P53 | p.R273H | Loss-of-function | 0 |
| T-47D | TP53 | P53 | p.L194F | Likely Loss-of-function | 0 |
| T-47D | PIK3CA | PI3K_c | p.H1047R | Gain-of-function | 1 |
