# Supplementary material for: Boolean modeling of breast cancer signaling pathways uncovers mechanisms of drug synergy
Source: PLoS One. 2024 Feb 23;19(2):e0298788. doi: 10.1371/journal.pone.0298788 (PMC10889607; doi:10.1371/journal.pone.0298788)
Supplement: S2 Table — (DOCX) [file pone.0298788.s003.docx]

**Table S2.** Examples of drug pairs with inconsistent synergy scores across five cell lines.

| Cell line | Drug pair | HSA model | LOEWE model | BLISS model | ZIP model |
| --- | --- | --- | --- | --- | --- |
| MCF-7 | ANASTROZOLE  & ARSENIC TRIOXIDE | −4.56 | −5.43 | 1.29 | 4.1 |
| T-47D | 5FU & ABT888 | 2.61 | −3.16 | 4.41 | 2.10 |
| MDA-MB-468 | BUSULFAN  & NSC-127716 | 3.53 | −2.61 | 0.1 | 0.51 |
| MDA-MB-231 | BUSULFAN  &CISPLATINO | 3.60 | −26.2 | 3.36 | 1.39 |
| BT-549 | BUSULFAN  & ARSENIC TRIOXIDE | 1.44 | −26.14 | 1.1 | −0.8 |
