# Supplementary material for: Boolean modeling of breast cancer signaling pathways uncovers mechanisms of drug synergy
Source: PLoS One. 2024 Feb 23;19(2):e0298788. doi: 10.1371/journal.pone.0298788 (PMC10889607; doi:10.1371/journal.pone.0298788)
Supplement: S1 Fig — (DOCX) [file pone.0298788.s004.docx]

**
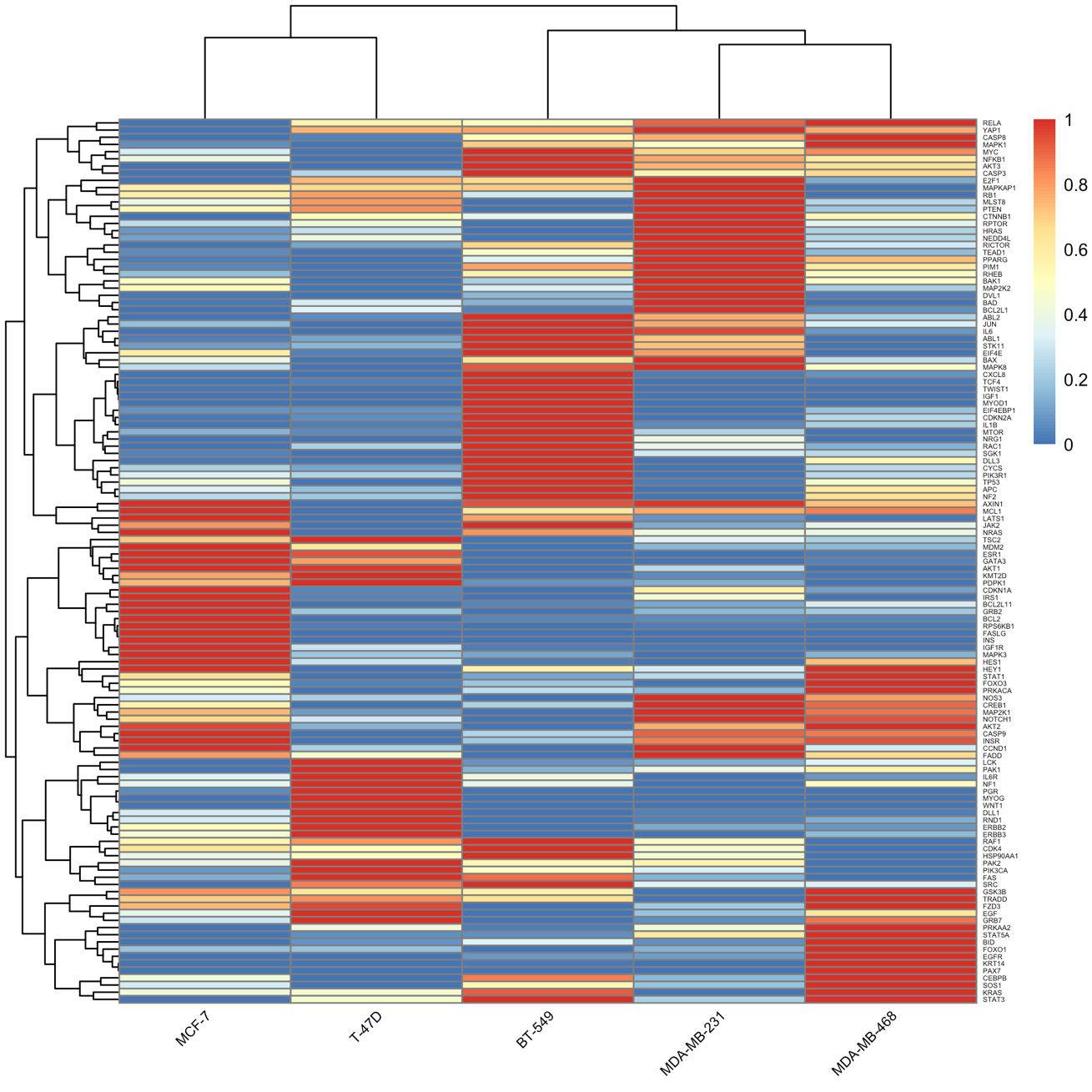
**

**S1 Fig.** Clustering analysis of gene expression profiles. The expression values were scaled with the Min-Max normalization method to obtain a value between 0 and 1 before clustering.
