# Supplementary figures and images for: Boolean modeling of breast cancer signaling pathways uncovers mechanisms of drug synergy
Source: PLoS One. 2024 Feb 23;19(2):e0298788. doi: 10.1371/journal.pone.0298788 (PMC10889607; doi:10.1371/journal.pone.0298788)

**
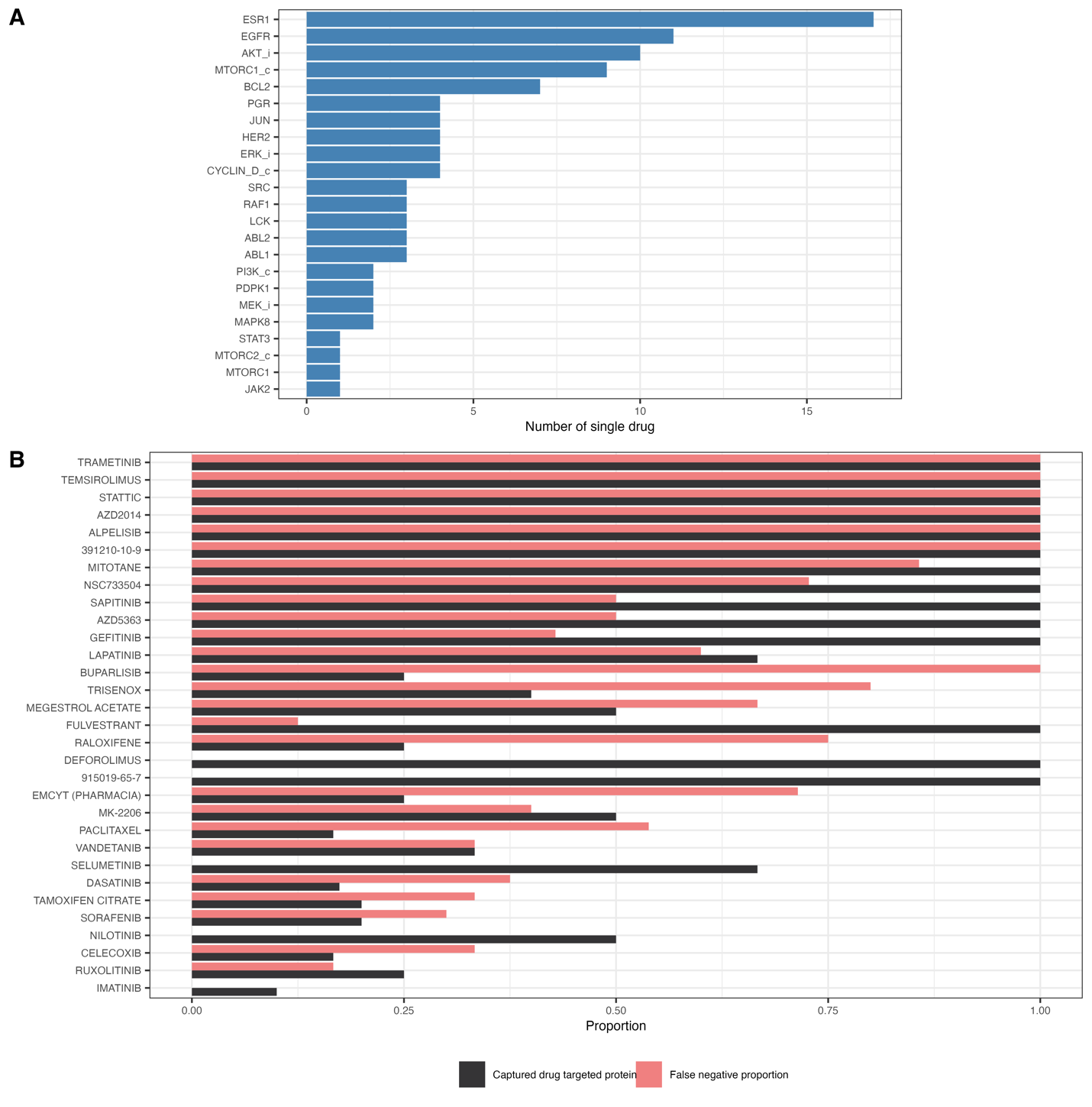
**

**S2 Fig.** The frequency of drug target proteins found in false-positive predictions.

Supplement: S2 Fig — (DOCX) [file pone.0298788.s005.docx]
