# Supplementary material for: Boolean modeling of breast cancer signaling pathways uncovers mechanisms of drug synergy
Source: PLoS One. 2024 Feb 23;19(2):e0298788. doi: 10.1371/journal.pone.0298788 (PMC10889607; doi:10.1371/journal.pone.0298788)
Supplement: Attachment — Submitted filename: Comments to the authors.pdf [file pone.0298788.s006.pdf]

Reviewer's Comments

Manuscript: Boolean modeling of breast cancer signaling pathways uncovers mechanisms of drug synergy

Comments to the Corresponding Author:

The authors aimed to model breast cancer signaling pathways using Boolean model, and make in-silico predictions to identify effective drug pairs. The manuscript is well presented and the methodology and results are explained very well. However, I believe the manuscript can be improved with a minor revision. I request the authors to consider the following suggestions and better their work.

In the methods section, few assumptions have been made without detailed justification. I request the authors to provide meaningful justifications for the following assumptions.

A. "An inhibitory regulator wins over all activating regulators." (Line 123). Please explain the biological rationality for this assumption.
B. "We assumed that the protein activities reached the steady state at the time step, whose slope of the entropy profile was the minimum." (Line 152). Please explain the logic of this assumption.
C. "Four doses (0, 0.25, 0.75, and 1) of each drug in combination were investigated for each drug pair. The doses represented the probability of the target-protein nodes being OFF or ON." (Line 172). Please explain the validity of this assumption of dosage-dependent intervention.
