# Supplementary material for: Boolean modeling of breast cancer signaling pathways uncovers mechanisms of drug synergy
Source: PLoS One. 2024 Feb 23;19(2):e0298788. doi: 10.1371/journal.pone.0298788 (PMC10889607; doi:10.1371/journal.pone.0298788)
Supplement: Attachment — Submitted filename: Response_to_Reviewers_PLOSONE2024_revision1.pdf [file pone.0298788.s007.pdf]

A point-by-point response to reviewers

Re: Boolean modeling of breast cancer signaling pathways uncovers mechanisms of drug synergy

Black = comments of editor or reviewers
Red = authors' replies
Black highlighted = changes made in the manuscript

Editor's comment

Thank you for submitting your manuscript to PLOS ONE. After careful consideration, we feel that it has merit but does not fully meet PLOS ONE's publication criteria as it currently stands. Therefore, we invite you to submit a revised version of the manuscript that addresses the points raised during the review process.
We would like to thank the editor and the reviewers for taking the time to provide feedback on our work. We have carefully considered your suggestions and made the necessary changes to the manuscript, as you will see below. We believe that these revisions have significantly strengthened the clarity and impact of our work, and we hope that you will find the revised version much improved.

Reviewers' comments

Reviewer #1:

The manuscript requires significant revisions; the authors must explain in detail how the Boolean models were constructed for each cell type and, what is being measured by the model, what the key takeaway is. From reading the manuscript at this stage, it needs to be clarified what is being measured or validated. The Drug synergy score, which is a pivotal part of this work, needs a more detailed explanation as well. The work, in general, has a lot of potential; there is the modeling of BC pathways, there is drug dosage consideration, and the model takes into account the dynamic behavior of the biological system. I encourage the authors to submit a significantly revised manuscript with more clarity. Following are my comments where the authors can improve their current work.
We would like to thank the reviewer for taking the time to review our work and providing valuable feedback. As you will see below, we have carefully considered the concerns raised and have significantly revised our manuscript. We hope that you will find the revised version to be much improved.

Page 2, Introduction: Why did the authors limit themselves to two drugs or drug pairs ? Is that usually the maximum number of targeted therapy drugs administered to patients? Please cite the reason along with the appropriate reference.
We limited our work to drug pairs because most of the experiments from the literature and the database (DrugComb) that we used to develop and validate our model are limited to only two drugs. However, our model can potentially simulate the effect of more than two drugs. In the revised manuscript, we have added this point to the Discussion.
Lines 443–448:

Our current study focused on simulating combinations of only two drugs because most drug-perturbed experiments involved only drug pairs. In addition, the HSA synergy scores from the DrugComb database to which we compared our synergy predictions were also limited to two drugs. However, cancer patients can be treated with more than two drugs if they offer greater efficacy (Xu et al., 2016), help to overcome drug resistance (Fisusi & Akala, 2019), and avoid drug toxicity (Nikanjam et al., 2017). It is worth noting that our simulation framework allows the simulation of more than two drug combinations, thereby providing further valuable predictions, which are planned for future investigation of the study.

Page 3, Introduction: Plos One has a readership of diverse backgrounds, so it will be appropriate for authors to explain drug synergy in detail. Authors should explain how drug synergy is measured in their domain of work. This is also not clarified later in the methods section.

We have added the definition and the calculations of drug synergy in detail in Lines 50–58:

When two drugs are combined, their effect may interact to enhance or reduce their effectiveness compared to when used separately, referred to as synergistic and antagonistic effects, respectively (Foucquier & Guedj, 2015). Several models have been proposed to quantify the degree of synergy, such as Highest Single Agent (HSA), Loewe, ZIP, and Bliss (Malyutina et al., 2019). These models share a common approach to calculating the synergy score S by determining the deviation of drug combination effect y_c from the expected effect y_e, $S = y_c - y_e$. Each model uses a different hypothesis for the expected effect calculation. For example, the HSA model assumes that the expected effect (y_e) of drug combination is equal to the effect of either drug A (y_A) or drug B (y_B), whichever is the greater. Then, the HSA synergy score (S_{HSA}) is calculated as $S_{HSA} = y_c - \max(y_A, y_B)$, where y_c is the effect of the drug combination. Positive and negative values of S_{HSA} indicate synergistic and antagonistic effects, respectively.

We also clarified how predicted drug synergy was calculated from our proposed model at Lines 252–263.

To calculate drug synergy scores from our model and compare them to the values reported in the DrugComb database, we proposed the calculation of the *PSS*. First, we categorized proteins in the model into onco- or tumor-suppressor proteins. Then, we calculated the *PSS* using the protein activities at the steady state, according to Eqs. 7 and 8:

$$PSS_{\text{onco-protein}} = \min(X_1^{SS}, X_2^{SS}) - X_{1+2}^{SS} \quad (7)$$

$$PSS_{\text{tumor-suppressor protein}} = Y_{1+2}^{SS} - \max(Y_1^{SS}, Y_2^{SS}), \quad (8)$$

where X^{SS} and Y^{SS} represent the steady-state protein activity of an oncoprotein and tumor-suppressor protein, respectively. The subscripts 1, 2, and 1+2 indicate the steady-state protein activity after being perturbed by drug 1, drug 2, and both, respectively. The calculation of *PSS*s for each dose followed the methodology for computing the highest single agent (HSA) synergy score. The value of the *PSS* is between −1 and +1, where the positive and negative values were interpreted as the drug pair having a synergistic and antagonistic effect on the protein level, respectively. The *PSS* of 0 indicated the additive effect. The *PSS* at the 75th percentile across all dose combinations was designated the consensus *PSS* for that protein. Finally, the *PSSs* of selected proteins were summed together to represent the predicted HSA synergy score for each drug pair.

Page 3, Introduction, Line 65: Please explain the reason why a Boolean framework was selected.
We now added at Lines 68–71:
Boolean modeling is one of the simplest mechanistic approaches, where kinetic parameters are not required. Thus, creating a larger protein signaling network with Boolean modeling is more feasible compared to kinetic-based models. As a result, the Boolean model can include more relevant proteins, such as drug target proteins (Karlebach & Shamir, 2008).

Page 3, Methods , Line 76-79: Why these cell lines and why 3 TNBC cell lines and 2 ER positive ? What impact does the unequal number of cell lines have on your findings ?
We have added at Lines 87–90:
These five cell lines are commonly used in breast cancer research to represent molecular subtypes of the ER-positive (MCF-7 and T-47D) and triple-negative (BT-549, MDA-MB-231, and MDA-MB-468) cancer (for example, see (Lim et al., 2020; Morotti et al., 2019; Sarmiento-Salinas et al., 2019; Shi et al., 2016)). The five cell lines are also experimentally reported in the DrugComb database with the highest numbers of tested drug pairs among other breast cancer cell lines (Zheng et al., 2021).

We agree with the reviewer that the currently selected cell lines represent unequal subtypes of cell lines (3 TNBC and 2 ER-positive) and cover only a small portion of breast cancer cell heterogeneity. More cell lines should be studied using the proposed framework of this work to strengthen the findings and ensure their generalizability to a broader range of cell types.
We have added this point to the Discussion at Lines 470–475.
It should be mentioned that the five cell lines currently selected for the study are not representative of all subtypes of breast cancer cells. Among the five cell lines, three are triple-negative breast cancer (TNBC), and two are estrogen receptor-positive (ER-positive). This limited selection of cell lines only covers a small portion of breast cancer cell heterogeneity. Therefore, it is essential to investigate more cell lines using the proposed framework of this study to ensure the generalizability of the findings to a broader range of cell types.

Page 4, Network Reconstruction, Line 96-99: Authors should clearly state if they chose all the pathways for breast cancer from Kegg. Furthermore, which proteins and interactions were picked from SIGNOR2.0 and SignaLink , should be mentioned in the manuscript text. Also, why did the authors not select pathways specific to ER+ and TNBC ?
We have selected all pathways related to breast cancer as specified by KEGG (https://www.genome.jp/pathway/hsa05224). We did not choose pathways specific to ER-positive and TNCB because we would like our framework to be expandable to other subtypes of breast cancer in the future.

Regarding which proteins and interactions were picked from SIGNOR2.0 and SignaLink, we revised our manuscript to clarify this.
Lines 111–121:
Breast cancer-related protein signaling pathways were retrieved from the KEGG database (https://www.genome.jp/pathway/hsa05224), which contains 76 proteins and 60 interactions (accessed on 1 March 2020). Another set of 41 proteins and an additional 176 interactions from the SIGNOR2.0 (https://signor.uniroma2.it/) and 12 interactions from SignaLink

(http://signalink.org/) databases (accessed on 1 January 2022) were combined. In addition, self-interactions (auto-activation) of nine ligand nodes (e.g., estrogen; ES and progesterone; PG) were included by assuming that these ligands are produced by cancer cells in an autocrine manner (Sporn & Roberts, 1985). The resulting protein signaling network comprises 117 nodes and 257 edges (Fig 2). The complete node and interaction list from KEGG, SIGNOR2.0, and SignaLink can be found in S1 File.

We have also analyzed the contributions of the proteins to the enrichment of the pathways by comparing the retrieved proteins from KEGG alone and when proteins from SIGNOR2.0 and SignaLink were included.
Lines 288–298:
Using the STRINGdb API (https://pypi.org/project/stringdb/), the majority of the original 76 signaling proteins from the KEGG database were significantly enriched in the PI3K-AKT (hsa04151: N=51 FDR=8.74×10^{-64}) and MTOR (hsa04150: N=29, FDR=7.05×10^{-50}) signaling pathways.

When 41 more nodes from the SIGNOR and SignaLink databases were included in the network, more pathways showed significant enrichment, e.g., apoptosis (hsa04210: N=21 from KEGG and 8 from SIGNOR and SignaLink, FDR=3.46×10^{-42}), ERBB (hsa04012: N=22 from KEGG and 8 from SIGNOR and SignaLink, FDR=4.04×10^{-48}), MAPK (hsa04010: N=22 from KEGG and 9 from SIGNOR and SignaLink, FDR=3.34×10^{-33}), and FOXO (hsa04068: N=29 from KEGG and 2 from SIGNOR and SignaLink, FDR=7.49×10^{-46}) signaling pathways.

Overall, the reconstructed protein signaling network comprehensively covered multiple breast cancer growth and proliferation pathways, including the PI3K-AKT, MTOR, apoptosis, ERBB, MAPK, and FOXO signaling pathways.

Page 4, Network Reconstruction Line 99: When the authors mention network, they should clarify what sort of network is it (Flow charts, boolean network etc).
We have now mentioned the network as the protein signaling network throughout the manuscript whenever appropriate.

Page 5, Generic Boolean Model, Line 113: Authors should explain in detail why a boolean modeling approach is appropriate for modeling protein activity.
We have now explained in Lines 68–71:
Boolean modeling is one of the simplest mechanistic approaches, where kinetic parameters are not required. Thus, creating a larger protein signaling network with Boolean modeling is more feasible compared to kinetic-based models. As a result, the Boolean model can include more relevant proteins, such as drug target proteins (Karlebach & Shamir, 2008).

Page 5, Generic Boolean Model, Line 123: Why were inhibitory regulators chosen to be modeled using an AND gate instead of an OR gate ? What is the biological significance.
The choice of using AND over OR was initially made for the generic model in a manner similar to the previous studies (Flobak et al., 2015; Niederdorfer et al., 2020; Tsirvouli et al., 2020).
This information is provided at Line 143:

These assumptions were initially made for the generic model in a manner similar to the previous studies (Flobak et al., 2015; Niederdorfer et al., 2020; Tsirvouli et al., 2020).

However, during the cell-line specific model construction, the Boolean rules of each cell line were manually modified to match model simulations with gene expression data and observed behaviors from drug-perturbed experiments.
The information is in Lines 181–185:
Each cell-line specific model (i.e., the generic model that incorporated genetic mutation and gene expression profiles) was simulated to generate protein activities at the steady state. The simulation results were compared to gene expression data and observed behaviors from drug-perturbed experiments of each respective cell line. During this step, we manually modified the Boolean rules of each cell-line specific model to capture observed behaviors derived from the experimental data.

Then, we provide one example of modifying an AND gate to an OR gate for inhibitory inhibitors at Lines 218–224:
In another instance, KMT2D, which is regulated positively and negatively by PAX7 and AKT, respectively, was described by a generic Boolean function

$$KMT2D\_c(t+1) = PAX7(t) \text{ AND NOT } AKT\_i(t) \quad (5)$$

The function was modified to

$$KMT2D\_c(t+1) = PAX7(t) \text{ OR NOT } AKT\_i(t) \quad (6)$$

in MCF-7 to reduce the contribution of AKT in inactivating KMT2D. The modification was made to match the simulation result with the observed expression data of KMT2D in MCF-7, which has a scaled value of 0.78 (out of the maximum value of 1.0). In contrast, for MDA-MB-231, the generic function in Eq. 5 was used as the scaled expression data of KMT2D in this cell line was 0.

Page 5, Cell Line Specific Model, Line 123: The authors need to be more specific about tailoring cell line-specific Boolean models. Did they add/remove pathways based on ER+ or TNBC? If so, which pathways were considered and which were removed from the original network?
We did not add or remove pathways when we created cell-line specific models. We manually modified logical rules (e.g., changing the rule from AND to OR, and vice versa) and by adding/removing edges to match model simulations with gene expression data and observed behaviors from 42 collected drug-perturbed experiments.

To better explain how the cell-line specific Boolean models were constructed, we have re-written the 'Cell-line specific models' section (Section 3 of Methods in the revised manuscript) and divided the section into three sub-sections:

3.1 Integrating cell line mutation profiles into the Boolean functions

3.2 Setting initial states of the cell lines based on gene expression profiles

3.3 Modifying the Boolean rules to match model simulations with gene expression data and observed behaviors from drug-perturbed experiments.

During Step 3.3, the Boolean rules of each cell-line specific model were manually modified to match model simulations with gene expression data and observed behaviors from 42 collected drug-perturbed experiments.
The information is in Lines 181–185:
Each cell-line specific model (i.e., the generic model that incorporated genetic mutation and gene expression profiles) was simulated to generate protein activities at the steady state. The simulation results were compared to gene expression data and observed behaviors from drug-perturbed experiments of each respective cell line. During this step, we manually modified the Boolean rules of each cell-line specific model to capture observed behaviors derived from the experimental data.

At Lines 204–227:
We collected 42 drug-treatment experiments on the five cell lines from published literature. We conducted a corresponding simulation for each experiment by fixing the Boolean values of the drug target nodes to 0 or 1, depending on whether the drug was an antagonist or agonist. We then compared the simulated response of the model to the observed behavior of the cell lines in the experiments.

During this step, inconsistency between simulations and experimental observations was addressed by manually modifying logical rules (e.g., changing the rule from AND to OR, and vice versa) and by adding/removing edges until the Boolean model of each cell line reproduced the gene expression and drug-treatment experiment observations. The adjustments were informed based on the literature as listed in S1 File.

For example, the generic Boolean function of MTORC1

$$MTORC1\_c(t+1) = RHEB(t) \quad (3)$$

was modified into the ER-positive specific Boolean function

$$MTORC1\_c(t+1) = RHEB(t) \text{ or } NRG1(t) \text{ or } IGF1(t) \text{ or } PIM(t) \quad (4)$$

because, in the generic Boolean model, MTORC1 was activated only by RHEB, which is a downstream target of AKT (Eq. 3). However, the regulatory function of MTORC1 of T-47D and MCF-7 further included NRG1, IGF1, and PIM (Eq. 4). This modification was based on supporting evidence showing that the presence of NRG1, IGF1, or PIM could maintain the MTORC1 activity even when an inhibitor suppressed the AKT activity (Elkabets et al., 2013; Le et al., 2016).

In another instance, KMT2D, which is regulated positively and negatively by PAX7 and AKT, respectively, was described by a generic Boolean function

$$KMT2D\_c(t+1) = PAX7(t) \text{ AND NOT } AKT\_i(t) \quad (5)$$

The function was modified to

$$KMT2D\_c(t+1) = PAX7(t) \text{ OR NOT } AKT\_i(t) \quad (6)$$

in MCF-7 to reduce the contribution of AKT in inactivating KMT2D. The modification was made to match the simulation result with the observed expression data of KMT2D in MCF-7, which has

a scaled value of 0.78 (out of the maximum value of 1.0). In contrast, for MDA-MB-231, the generic function in Eq. 5 was used as the scaled expression data of KMT2D in this cell line was 0.

The complete logical rules for each cell-line specific model are listed in S1 File. The 42 experiments whose information was used to modify and validate the Boolean rules will be discussed further in the Results section (Table 2 below).

Page 6, Cell Line Specific Model, Line 144: It is unclear what the Boolean model is measuring and what is being validated. The authors need to expand their model explanation in the previous and current section.

In the revised manuscript, we have added more information to the paragraph describing the overall workflow of the current work at Lines 95–101:

Each cell-line specific model was simulated to obtain steady-state protein activities under unperturbed and drug-perturbed conditions. The protein activities of each cell line under unperturbed conditions were compared to their respective gene expression data. The results of the drug perturbation simulations were compared to the observed responses of the cell lines to drug treatments, which were collected from the literature. The Boolean rules of each cell-line specific model were manually modified during this step to match model simulations with gene expression data and observed behaviors from drug-perturbed experiments

We also have added to Lines 181–185 (Section Methods):

Each cell-line specific model (i.e., the generic model that incorporated genetic mutation and gene expression profiles) was simulated to generate protein activities at the steady state. The simulation results were compared to gene expression data and observed behaviors from drug-perturbed experiments of each respective cell line. During this step, we manually modified the Boolean rules of each cell-line specific model to capture observed behaviors derived from the experimental data.

and to Lines 301–306 (Section Results):

The five cell-line specific models were simulated with and without drug perturbations to obtain protein activities at the steady state. The unperturbed protein activities from each cell line were compared to their respective cell lines' actual gene expression data retrieved from the Cell Model Passports database. The drug perturbation simulations were compared to observed responses of the cell lines to drug treatments collected from 42 experiments. The results of these simulations demonstrated that the models could capture the unique characteristics of each cell line and their responses to drug perturbations.

Page 7 , Cell Line Specific Model, Line 162: Can the authors provide an example from their simulation of these incosistencies ?

We added an example of these inconsistencies to Lines 212–217:

For example, the generic Boolean function of MTORC1

$$MTORC1\_c(t+1) = RHEB(t) \qquad (3)$$

was modified into the ER-positive specific Boolean function

$$MTORC1\_c(t+1) = RHEB(t) \text{ or } NRG1(t) \text{ or } IGF1(t) \text{ or } PIM(t) \qquad (4)$$

because, in the generic Boolean model, MTORC1 was activated only by RHEB, which is a downstream target of AKT (Eq. 3). However, the regulatory function of MTORC1 of T-47D and MCF-7 further included NRG1, IGF1, and PIM (Eq. 4). This modification was based on supporting evidence showing that the presence of NRG1, IGF1, or PIM could maintain the MTORC1 activity even when an inhibitor suppressed the AKT activity (Elkabets et al., 2013; Le et al., 2016).

Page 7, Drug Combination Simulations, Line 172: How did the authors normalize standard dosage information to 0, 0.25,0.75 and 1 ?
Why only these four discrete probablities instead of a continous distribution ?
We added our rationale of normalizing drug dosage to 0–1
Lines 244–249:
Implementing drug effects on protein targets as a probability between 0 and 1 reflects the concentration ranges of monotherapy that cover the minimum and the maximum effect of drugs as implemented in the drug combination experiments (Houweling et al., 2023; Jaaks et al., 2022; Mäkelä et al., 2021). We assume that a drug concentration with the maximum effect (equivalent to a dose of 1.0 in our simulation) would fully inhibit the protein targets with a 100% chance. Similarly, a drug concentration at half the maximum effect (equivalent to a dose of 0.5 in our simulation) would inhibit the protein targets with a 50% chance.

We selected only four discrete doses instead of a continuous distribution to reduce the computational time required to simulate the model. We now clarified this at Lines 249–251:
However, as simulating various dose-response profiles can be computationally intensive, we have selected to use only four doses for each drug. As a result, the simulations produced 4×4 protein activity profiles per drug pair per cell line.

Page 8, Drug Combination Simulations, Line 185: The authors should clearly define the meaning of PSS and its range of value.
The *PSS*s determine the synergistic or antagonistic effect of drug pairs on the protein level. The range of a *PSS* is between −1 and +1. The *PSS*s of selected proteins were summed together to calculate the predicted HSA synergy score for each drug pair.
We have added this information to Lines 252–263:
To calculate drug synergy scores from our model and compare them to the values reported in the DrugComb database, we proposed the calculation of the *PSS*. First, we categorized proteins in the model into onco- or tumor-suppressor proteins. Then, we calculated the *PSS* using the protein activities at the steady state, according to Eqs. 7 and 8:

$$PSS_{\text{onco-protein}} = \min(X_1^{\text{SS}}, X_2^{\text{SS}}) - X_{1+2}^{\text{SS}} \quad (7)$$
$$PSS_{\text{tumor-suppressor protein}} = Y_{1+2}^{\text{SS}} - \max(Y_1^{\text{SS}}, Y_2^{\text{SS}}), \quad (8)$$

where X^{SS} and Y^{SS} represent the steady-state protein activity of an oncoprotein and tumor-suppressor protein, respectively. The subscripts 1, 2, and 1+2 indicate the steady-state protein activity after being perturbed by drug 1, drug 2, and both, respectively. The calculation of *PSS*s for each dose followed the methodology for computing the highest single agent (HSA) synergy score. The value of the *PSS* is between −1 and +1, where the positive and negative values were interpreted as the drug pair having a synergistic and antagonistic effect on the protein level, respectively. The *PSS* of 0 indicated the additive effect. The *PSS* at the 75th percentile across all

dose combinations was designated the consensus *PSS* for that protein. Finally, the *PSSs* of selected proteins were summed together to represent the predicted HSA synergy score for each drug pair.

Reviewer #2:

The authors are trying to tackle an important problem of find an ideal drug combination using synergistic scores.

1) The authors say that their approach shares similarity with Tsirvouli et al. (2020) and that they have extended it for 5 cell lines. It will be nice to know what other distinctions and improvements the study has made.

We now mentioned the main distinctions of our work from Tsirvouli et al.'s 2020 paper in Lines 427–433:

Our approach shares similarities with the methodology employed by Tsirvouli et al. (2020) but extends the analysis to five breast cancer cell lines covering both ER-positive and triple-negative subtypes of breast cancers. In addition, a unique feature in the current study is the calculation of the protein synergy scores (*PSS*s) that determine the effect of drug combinations on individual proteins' activities. The calculation allowed the synergistic effects of drug pairs to be decomposed into the contributions made by individual proteins, thereby enabling the interpretation of the mechanism of the synergy through the profile of protein activities.

2) It will be easy for reader if the authors can show the Boolean model equivalent for Fig. 2.

The Boolean model equivalent to Fig 2 can be found in S1 File. In the main text, we give an example of a Boolean rule in Lines 144–148:

For instance, AKT is recruited by PIP3 at the plasma membrane. Subsequently, AKT is phosphorylated twice by MTORC2 and PDPK1 at the S473 and T308 sites, respectively, to be fully activated (Manning & Toker, 2017). These interactions were described as

$$AKT\_i(t+1) = PIP3(t) \text{ AND } MTORC2\_c(t) \text{ AND } PDPK1(t)$$

The complete Boolean rules of the generic model (Fig 2) can be found in S1 File.

3) I feel details are missing on how to recreate the models the authors are proposing. This will make it challenging for others to replicate and build upon their studies.

We now provide codes and related data to reproduce the simulations on GitHub.

Lines 490–492:

Data availability

Programming script and related data required to reproduce the simulations are available at https://github.com/kittisaktaoma/BoolSyn/.

In addition, to better explain how the cell-line specific Boolean models were constructed, we have re-written the 'Cell-line specific models' section (Section 3 of Methods in the revised manuscript) and divided the section into three sub-sections:

3.1 Integrating cell line mutation profiles into the Boolean functions

3.2 Setting initial states of the cell lines based on gene expression profiles

3.3 Modifying the Boolean rules to match model simulations with gene expression data and observed behaviors from drug-perturbed experiments.

During Step 3.3, the Boolean rules of each cell-line specific model were manually modified to match model simulations with gene expression data and observed behaviors from 42 collected drug-perturbed experiments.
The information is in Lines 181–185:
Each cell-line specific model (i.e., the generic model that incorporated genetic mutation and gene expression profiles) was simulated to generate protein activities at the steady state. The simulation results were compared to gene expression data and observed behaviors from drug-perturbed experiments of each respective cell line. During this step, we manually modified the Boolean rules of each cell-line specific model to capture observed behaviors derived from the experimental data.

At Lines 204–227:
We collected 42 drug-treatment experiments on the five cell lines from published literature. We conducted a corresponding simulation for each experiment by fixing the Boolean values of the drug target nodes to 0 or 1, depending on whether the drug was an antagonist or agonist. We then compared the simulated response of the model to the observed behavior of the cell lines in the experiments.

During this step, inconsistency between simulations and experimental observations was addressed by manually modifying logical rules (e.g., changing the rule from AND to OR, and vice versa) and by adding/removing edges until the Boolean model of each cell line reproduced the gene expression and drug-treatment experiment observations. The adjustments were informed based on the literature as listed in S1 File.

For example, the generic Boolean function of MTORC1

$$MTORC1\_c(t+1) = RHEB(t) \quad (3)$$

was modified into the ER-positive specific Boolean function

$$MTORC1\_c(t+1) = RHEB(t) \text{ or } NRG1(t) \text{ or } IGF1(t) \text{ or } PIM(t) \quad (4)$$

because, in the generic Boolean model, MTORC1 was activated only by RHEB, which is a downstream target of AKT (Eq. 3). However, the regulatory function of MTORC1 of T-47D and MCF-7 further included NRG1, IGF1, and PIM (Eq. 4). This modification was based on supporting evidence showing that the presence of NRG1, IGF1, or PIM could maintain the MTORC1 activity even when an inhibitor suppressed the AKT activity (Elkabets et al., 2013; Le et al., 2016).

In another instance, KMT2D, which is regulated positively and negatively by PAX7 and AKT, respectively, was described by a generic Boolean function

$$KMT2D\_c(t+1) = PAX7(t) \text{ AND NOT } AKT\_i(t) \quad (5)$$

The function was modified to

$$KMT2D\_c(t+1) = PAX7(t) \text{ OR NOT } AKT\_i(t) \quad (6)$$

in MCF-7 to reduce the contribution of AKT in inactivating KMT2D. The modification was made to match the simulation result with the observed expression data of KMT2D in MCF-7, which has

a scaled value of 0.78 (out of the maximum value of 1.0). In contrast, for MDA-MB-231, the generic function in Eq. 5 was used as the scaled expression data of KMT2D in this cell line was 0.

The complete logical rules for each cell-line specific model are listed in S1 File. The 42 experiments whose information was used to modify and validate the Boolean rules will be discussed further in the Results section (Table 2 below).

4) The authors say it is a Boolean model but then they refer to normalized/scaled expression values between 0 and 1. They should clarify this in the text.

We averaged the Boolean values (0 or 1) of each protein from multiple asynchronous update simulations to obtain the protein activities (values ranging between 0 and 1).

We now have clarified this in Lines 186–193:

To compare protein activities simulated from the Boolean model (values of 0 or 1) to gene expression data (values obtained from the Cell Model Passports database and scaled to range between 0 and 1), we performed model simulation for 5000-time steps using a uniform asynchronous update scheme. In this scheme, only one node from all nodes that could change their state was selected with equal probability among others to change per time step. One hundred repeats were simulated for each cell-line specific model. The resulting Boolean values (0 or 1) of each protein from all repeats were averaged over the same time steps to obtain the protein activities (values ranging between 0 and 1), and the protein activities at the steady state were compared to the retrieved gene expression data from the Cell Model Passports database.

5) They use asynchronous updates for their model. I believe they should look into literature and have an idea of time-scales of the different reactions and apply update times accordingly. They can incorporate a delay counter into their model.

We thank the reviewer for the suggestion. Incorporating time scales of different reactions will make the model more realistic and enhance the model's accuracy. We would like to explore this suggestion in future work. We added this point to the Discussion at Lines 468–470:

The use of asynchronous updates in the current study did not consider the varying time scales of different reactions (e.g., protein synthesis versus phosphorylation/dephosphorylation). Incorporating this aspect into future versions of the model may enhance the simulation's accuracy.

6) I am not sure how acceptable is the idea that 0.25 of a drug has a 25% chance of inhibiting the protein.

We added our rationale of normalizing drug dosage to a probability between 0 and 1.

Lines 244–251

Implementing drug effects on protein targets as a probability between 0 and 1 reflects the concentration ranges of monotherapy that cover the minimum and the maximum effect of drugs as implemented in the drug combination experiments (Houweling et al., 2023; Jaaks et al., 2022; Mäkelä et al., 2021). We assume that a drug concentration with the maximum effect (equivalent to a dose of 1.0 in our simulation) would fully inhibit the protein targets with a 100% chance. Similarly, a drug concentration at half the maximum effect (equivalent to a dose of 0.5 in our simulation) would inhibit the protein targets with a 50% chance. However, as simulating various dose-response profiles can be computationally intensive, we have selected to use only four doses for each drug. As a result, the simulations produced 4×4 protein activity profiles per drug pair per cell line.

7) There is no explanation for Fig. 3 and how it correlates with the published literature. There is no interpretation of the output by the authors.

We have added more detail in the text at Lines 309–317:

We conducted simulations for each cell-line specific Boolean model without drug perturbations to derive the steady-state protein activities. The simulated steady-state protein activities exhibited a reasonable level of correlation with the actual gene expression data, with Pearson's correlation coefficients ranging between 0.67 and 0.85. MDA-MB-231 and T-47D were the cell lines from triple-negative and ER-positive subtypes with the highest correlation at 0.85 and 0.75, respectively. An identical Pearson's correlation at 0.67 was observed for MDA-MB-468 and BT-549, whereas MCF-7 was achieved at 0.68. When we performed clustering analysis, combining these simulated steady-state protein activities with the actual gene expression data, the results accurately segregated into the ER-positive (T-47D and MCF-7) and triple-negative (BT-549, MDA-MB-231, and MDA-MB-468) sub-groups, as demonstrated in Fig 3. This suggests that our cell-line specific models behaved similarly to their unperturbed cell-line behaviors.

I believe the authors have to rework the manuscript to make it easily readable as well as explain their algorithms and figures better.

We would like to thank the reviewer for taking the time to review our work and providing valuable feedback. We hope you will find the revised version much more easily readable.

Reviewer #3:

Comments to the Corresponding Author:

The authors aimed to model breast cancer signaling pathways using Boolean model, and make in-silico predictions to identify effective drug pairs. The manuscript is well presented and the methodology and results are explained very well. However, I believe the manuscript can be improved with a minor revision. I request the authors to consider the following suggestions and better their work.

In the methods section, few assumptions have been made without detailed justification. I request the authors to provide meaningful justifications for the following assumptions.

A. “An inhibitory regulator wins over all activating regulators.” (Line 123). Please explain the biological rationality for this assumption.

The assumption of “An inhibitory regulator wins over all activating regulators” was initially made for the generic model in a manner similar to the previous studies (Flobak et al., 2015; Niederdorfer et al., 2020; Tsirvouli et al., 2020).

This information is provided at Line 143:

These assumptions were initially made for the generic model in a manner similar to the previous studies (Flobak et al., 2015; Niederdorfer et al., 2020; Tsirvouli et al., 2020).

However, during the cell-line specific model construction, the Boolean rules of each cell line were manually modified to match model simulations with gene expression data and observed behaviors from drug-perturbed experiments.

The information is in Lines 181–185:

Each cell-line specific model (i.e., the generic model that incorporated genetic mutation and gene expression profiles) was simulated to generate protein activities at the steady state. The simulation results were compared to gene expression data and observed behaviors from drug-perturbed experiments of each respective cell line. During this step, we manually modified the Boolean rules of each cell-line specific model to capture observed behaviors derived from the experimental data.

Then, we provide one example of modifying the assumption of 'inhibitory regulators win over activating regulators' to 'activating regulators win over inhibitory regulators' at Lines 218–224:
In another instance, KMT2D, which is regulated positively and negatively by PAX7 and AKT, respectively, was described by a generic Boolean function

$$KMT2D\_c(t+1) = PAX7(t) \text{ AND NOT } AKT\_i(t) \quad (5)$$

The function was modified to

$$KMT2D\_c(t+1) = PAX7(t) \text{ OR NOT } AKT\_i(t) \quad (6)$$

in MCF-7 to reduce the contribution of AKT in inactivating KMT2D. The modification was made to match the simulation result with the observed expression data of KMT2D in MCF-7, which has a scaled value of 0.78 (out of the maximum value of 1.0). In contrast, for MDA-MB-231, the generic function in Eq. 5 was used as the scaled expression data of KMT2D in this cell line was 0.

B. "We assumed that the protein activities reached the steady state at the time step, whose slope of the entropy profile was the minimum." (Line 152). Please explain the logic of this assumption.
We added the explanation for this assumption in Lines 193–201:
The time step at which the steady state of the protein activities was reached was determined by entropy $H(t)$ (Stoll et al., 2012):

$$H(t) = -\sum_{S} \mathrm{P}_t(S)\log_2(\mathrm{P}_t(S)), \quad (2)$$

where $\mathrm{P}_t(S)$ is the probability the state S is observed over time step 0 to t. The entropy was calculated across the simulation time step (t = 0 to 5000) to obtain the entropy profile. A higher entropy value indicates a higher impurity level in the system (i.e., various states of protein activities are observed over time t). When a particular state of protein activities becomes dominant over time, indicating the steady state, the entropy level approaches 0 as the probability of observing the steady state is near 1, i.e., $\lim_{\mathrm{P}_t(S) \to 1} \log_2(\mathrm{P}_t(S)) = 0$. However, it may take a long computational time to simulate the model until the system is completely homogenous with a steady state. Therefore, we assume that the protein activities have reached a steady state when the slope of the entropy profile is at its minimum.

C. "Four doses (0, 0.25, 0.75, and 1) of each drug in combination were investigated for each drug pair. The doses represented the probability of the target-protein nodes being OFF or ON." (Line 172). Please explain the validity of this assumption of dosage-dependent Intervention.
We added our rationale of normalizing drug dosage to a probability between 0 and 1.
Lines 244–251:

Implementing drug effects on protein targets as a probability between 0 and 1 reflects the concentration ranges of monotherapy that cover the minimum and the maximum effect of drugs as implemented in the drug combination experiments (Houweling et al., 2023; Jaaks et al., 2022; Mäkelä et al., 2021). We assume that a drug concentration with the maximum effect (equivalent to a dose of 1.0 in our simulation) would fully inhibit the protein targets with a 100% chance. Similarly, a drug concentration at half the maximum effect (equivalent to a dose of 0.5 in our simulation) would inhibit the protein targets with a 50% chance. However, as simulating various dose-response profiles can be computationally intensive, we have selected to use only four doses for each drug. As a result, the simulations produced 4×4 protein activity profiles per drug pair per cell line.

We would like to thank the reviewer for the valuable suggestions. We hope the reviewer will find our revisions to be much improved.

References

Elkabets, M., Vora, S., Juric, D., Morse, N., Mino-Kenudson, M., Muranen, T., Tao, J., Campos, A. B., Rodon, J., Ibrahim, Y. H., Serra, V., Rodrik-Outmezguine, V., Hazra, S., Singh, S., Kim, P., Quadt, C., Liu, M., Huang, A., Rosen, N., … Baselga, J. (2013). mTORC1 inhibition is required for sensitivity to PI3K p110α inhibitors in PIK3CA-mutant breast cancer. *Science Translational Medicine*, *5*(196), 196ra99. https://doi.org/10.1126/scitranslmed.3005747

Fisusi, F. A., & Akala, E. O. (2019). Drug combinations in breast cancer therapy. *Pharmaceutical Nanotechnology*, *7*(1), 3–23. https://doi.org/10.2174/2211738507666190122111224

Flobak, Å., Baudot, A., Remy, E., Thommesen, L., Thieffry, D., Kuiper, M., & Lægreid, A. (2015). Discovery of drug synergies in gastric cancer cells predicted by logical modeling. *PLoS Computational Biology*, *11*(8), 1–20. https://doi.org/10.1371/journal.pcbi.1004426

Foucquier, J., & Guedj, M. (2015). Analysis of drug combinations: current methodological landscape. *Pharmacology Research & Perspectives*, *3*(3), e00149. https://doi.org/10.1002/prp2.149

Houweling, M., Giczewska, A., Abdul, K., Nieuwenhuis, N., Küçükosmanoglu, A., Pastuszak, K., Buijsman, R. C., Wesseling, P., Wedekind, L., Noske, D., Supernat, A., Bailey, D., Watts, C., Wurdinger, T., & Westerman, B. A. (2023). Screening of predicted synergistic multi-target therapies in glioblastoma identifies new treatment strategies. *Neuro-Oncology Advances*, *5*(1), vdad073. https://doi.org/10.1093/noajnl/vdad073

Jaaks, P., Coker, E. A., Vis, D. J., Edwards, O., Carpenter, E. F., Leto, S. M., Dwane, L., Sassi, F., Lightfoot, H., Barthorpe, S., van der Meer, D., Yang, W., Beck, A., Mironenko, T., Hall, C., Hall, J., Mali, I., Richardson, L., Tolley, C., … Garnett, M. J. (2022). Effective drug combinations in breast, colon and pancreatic cancer cells. *Nature*, *603*(7899), 166–173. https://doi.org/10.1038/s41586-022-04437-2

Karlebach, G., & Shamir, R. (2008). Modelling and analysis of gene regulatory networks. *Nature Reviews Molecular Cell Biology*, *9*(10), 770–780. https://doi.org/10.1038/nrm2503

Le, X., Antony, R., Razavi, P., Treacy, D. J., Luo, F., Ghandi, M., Castel, P., Scaltriti, M., Baselga, J., & Garraway, L. A. (2016). Systematic functional characterization of resistance to PI3K inhibition

in breast cancer. *Cancer Discovery*, *6*(10), 1134–1147. https://doi.org/10.1158/2159-8290.CD-16-0305

Lim, D., Cho, J. G., Yun, E., Lee, A., Ryu, H.-Y., Lee, Y. J., Yoon, S., Chang, W., Lee, M.-S., Kwon, B. S., & Kim, J. (2020). MicroRNA 34a-AXL axis regulates vasculogenic mimicry formation in breast cancer cells. *Genes*, *12*(1). https://doi.org/10.3390/genes12010009

Mäkelä, P., Zhang, S. M., & Rudd, S. G. (2021). Drug synergy scoring using minimal dose response matrices. *BMC Research Notes*, *14*(1), 1–6. https://doi.org/10.1186/s13104-021-05445-7

Malyutina, A., Majumder, M. M., Wang, W., Pessia, A., Heckman, C. A., & Tang, J. (2019). Drug combination sensitivity scoring facilitates the discovery of synergistic and efficacious drug combinations in cancer. *PLoS Computational Biology*, *15*(5), e1006752. https://doi.org/10.1371/journal.pcbi.1006752

Manning, B. D., & Toker, A. (2017). AKT/PKB Signaling: Navigating the network. *Cell*, *169*(3), 381–405. https://doi.org/10.1016/j.cell.2017.04.001

Morotti, M., Bridges, E., Valli, A., Choudhry, H., Sheldon, H., Wigfield, S., Gray, N., Zois, C. E., Grimm, F., Jones, D., Teoh, E. J., Cheng, W.-C., Lord, S., Anastasiou, D., Haider, S., McIntyre, A., Goberdhan, D. C. I., Buffa, F., & Harris, A. L. (2019). Hypoxia-induced switch in SNAT2/SLC38A2 regulation generates endocrine resistance in breast cancer. *Proceedings of the National Academy of Sciences of the United States of America*, *116*(25), 12452–12461. https://doi.org/10.1073/pnas.1818521116

Niederdorfer, B., Touré, V., Vazquez, M., Thommesen, L., Kuiper, M., Lægreid, A., & Flobak, Å. (2020). Strategies to enhance logic modeling-based cell line-specific drug synergy prediction. *Frontiers in Physiology*, *11*, 1–16. https://doi.org/10.3389/fphys.2020.00862

Nikanjam, M., Liu, S., Yang, J., & Kurzrock, R. (2017). Dosing three-drug combinations that include targeted anti-cancer agents: analysis of 37,763 patients. *The Oncologist*, *22*(5), 576–584. https://doi.org/10.1634/theoncologist.2016-0357

Sarmiento-Salinas, F. L., Delgado-Magallón, A., Montes-Alvarado, J. B., Ramírez-Ramírez, D., Flores-Alonso, J. C., Cortés-Hernández, P., Reyes-Leyva, J., Herrera-Camacho, I., Anaya-Ruiz, M., Pelayo, R., Millán-Pérez-Peña, L., & Maycotte, P. (2019). Breast cancer subtypes present a differential production of reactive oxygen species (ROS) and susceptibility to antioxidant treatment. *Frontiers in Oncology*, *9*, 480. https://doi.org/10.3389/fonc.2019.00480

Shi, Y., Yu, Y., Wang, Z., Wang, H., Bieerkehazhi, S., Zhao, Y., Suzuk, L., & Zhang, H. (2016). Second-generation proteasome inhibitor carfilzomib enhances doxorubicin-induced cytotoxicity and apoptosis in breast cancer cells. *Oncotarget*, *7*(45), 73697–73710. https://doi.org/10.18632/oncotarget.12048

Sporn, M. B., & Roberts, A. B. (1985). Autocrine growth factors and cancer. *Nature*, *313*(6005), 745–747. https://doi.org/10.1038/313745a0

Stoll, G., Viara, E., Barillot, E., & Calzone, L. (2012). Continuous time boolean modeling for biological signaling: application of Gillespie algorithm. *BMC Systems Biology*, *6*, 1–18. https://doi.org/10.1186/1752-0509-6-116

Tsirvouli, E., Touré, V., Niederdorfer, B., Vázquez, M., Flobak, Å., & Kuiper, M. (2020). A middle-out modeling strategy to extend a colon cancer logical model improves drug synergy predictions in epithelial-derived cancer cell lines. *Frontiers in Molecular Biosciences*, *7*, 1–19. https://doi.org/10.3389/fmolb.2020.502573

Xu, L., Wu, X., Hu, C., Zhang, Z., Zhang, L., Liang, S., Xu, Y., & Zhang, F. (2016). A meta-analysis of combination therapy versus single-agent therapy in anthracycline- and taxane-pretreated metastatic breast cancer: results from nine randomized Phase III trials. *OncoTargets and Therapy*, *9*, 4061–4074. https://doi.org/10.2147/OTT.S101423

Zheng, S., Aldahdooh, J., Shadbahr, T., Wang, Y., Aldahdooh, D., Bao, J., Wang, W., & Tang, J. (2021). DrugComb update: a more comprehensive drug sensitivity data repository and analysis portal. *Nucleic Acids Research*, *49*, W174–W184. https://doi.org/10.1093/nar/gkab438
